# Treatment failure in hyperthyroid cats after radioiodine (I‐131) injection

**DOI:** 10.1111/jvim.16161

**Published:** 2021-05-17

**Authors:** Deirdre Mullowney, Yu‐Mei Chang, Barbara Glanemann, Harriet M. Syme

**Affiliations:** ^1^ Department of Veterinary Clinical Sciences Royal Veterinary College, University of London Hertfordshire AL9 7TA United Kingdom; ^2^ Research Support Office The Royal Veterinary College London United Kingdom

**Keywords:** euthyroid, feline, persistent hyperthyroidism, thyroid gland

## Abstract

**Background:**

There is limited published information on the outcome for cats where total thyroxine concentration (TT4) remains elevated after treatment with radioactive iodine (RAI).

**Objective:**

To determine the frequency of, and predictors for, subsequent treatment failure in cats for which TT4 remains elevated at hospital discharge, and to report clinical outcomes for cats requiring repeat treatment.

**Animals:**

One hundred twenty‐one cats with TT4 ≥40 nmol/L after treatment with RAI (out of an original, treated study sample of 959 cats).

**Methods:**

Retrospective study. Data regarding signalment, weight, TT4 concentration (before RAI treatment, at discharge, and percentage change), day of sampling, and I‐131 dose were acquired. Logistic regression was performed to evaluate predictors of treatment failure.

**Results:**

In the 87 cats for which classification was possible, 35 (40%) became euthyroid without further treatment. All TT4 variables and weight normalized RAI dose were independently predictive of subsequent treatment failure. In multivariate analysis, TT4 concentration at discharge (*P* < .001) and weight normalized RAI dose (*P* = .04) remained in the final model. All 28 cats with TT4 concentration ≥150 nmol/L at discharge ultimately failed treatment, compared with 13/40 (32.5%) and 11/19 (57.9%) cats with TT4 concentrations of 40‐100 nmol/L and 100‐150 nmol/L, respectively. Of the 52 cats that failed treatment, 14 were subsequently managed medically, 12 underwent thyroidectomy (4 with carcinoma), 14 had repeat RAI treatment which was successful in 12/14 (86%) cats, and 13 had no further treatment.

**Conclusions and Clinical Importance:**

Cats with TT4 >150 nmol/L at discharge after RAI might be candidates for immediate repeat treatment.

**Abbreviations**
RAI: radioiodine treatment
TT4: total thyroxine concentration

## 1 INTRODUCTION

Hyperthyroidism in cats is a commonly diagnosed endocrinopathy with an overall prevalence of 2.4% in England which increases to 8.7% in cats aged 10 years or older.^1^ Radioactive iodine (RAI)] is generally considered the optimal treatment for hyperthyroidism in cats.^2^ The goal of RAI treatment is to restore euthyroidism while avoiding the development of hypothyroidism. The response to RAI treatment is rapid with 84.7% (444/524) cats having total thyroxine concentration (TT4) within or below reference range by the time of hospital discharge and TT4 is within or below the reference range in 98.5% of cats 6 months later.^3^ The development of iatrogenic hypothyroidism, after treatment with RAI, has been linked to the development of azotemia and reduced survival time.^4^ Avoidance of hypothyroidism has led to increasing interest in treatment with lower doses of RAI but this has the potential to increase the risk of treatment failure, at least in humans.^5^ Several studies have assessed the ideal dosing protocol for RAI.^6^, ^7^ A protocol for low‐dose (74 MBq or 2 mCi) treatment of hyperthyroidism has a high success rate for resolution of hyperthyroidism overall, however, the power of the study to detect a decrease in effectiveness with the lower treatment dose was only 5.5%, and several cats in that study had “high‐normal” thyroxine concentration (>3 μg/dL or >38 nmol/L) after treatment.^8^

In many localities where short‐hospital stays after RAI treatment are permitted, evaluation of the success of treatment is not performed until a month or more after discharge from the hospital. In the United Kingdom, regulation of radioisotope use in veterinary medicine is in general more stringent and cats are hospitalized for longer after RAI treatment. This means that initial evaluation is performed before the cat is discharged from the hospital, 2‐3 weeks after injection of RAI.

The greatest reduction in TT4 concentration occurs in the first month after treatment with RAI, although TT4 concentration can continue to decline for up to 6 months.^9^, ^10^, ^11^ If the TT4 concentration is high at the time of discharge it would be useful to know whether it is likely to continue to decrease resulting in the treatment being considered a success, or whether repeat treatment of the cat is necessary. If repeat treatment is required it would also be useful to know the likelihood of treatment success with a second injection of RAI, or with other types of therapy (surgical or medical management).

The purpose of this study was to report outcomes for cats that had elevated TT4 concentration at the time of discharge from hospital after RAI treatment of hyperthyroidism and to assess for predictors of ultimate treatment failure in these cats which might assist in clinical decision making for these cats. Another aim of this study was to report outcomes for cats that were retreated with RAI after the failure of initial treatment with RAI.

## 2 MATERIALS AND METHODS

### 2.1 Case selection

The study was approved by the local clinical research ethical review board (URN: SR2019‐0210). Records for all hyperthyroid cats referred for RAI treatment at the Queen Mother Hospital for Animals from January 2007 to October 2018 were reviewed in this retrospective study. Primary care practices were contacted for additional follow‐up data (further treatment, subsequent TT4 concentration measurements, and survival status) for cats in which TT4 concentration had not normalized at the time of discharge from the hospital. Inclusion criteria included any cat which underwent RAI treatment and had a TT4 concentration >40 nmol/L at the time of discharge from hospital (15 or 23 days after treatment). Data retrieved from medical records included breed, age, sex, bodyweight, TT4 concentration before RAI treatment, stay duration, and TT4 concentration at discharge. Total thyroxine concentration before RAI treatment was defined as TT4 concentration measured on admission to hospital for RAI treatment. The duration of stay was influenced when the TT4 concentration at discharge was measured. The TT4 concentration at discharge sample was obtained 23 days after RAI injection in cats described as “long stay” and 15 days after RAI injection in cats described as “short stay.” All cats treated with RAI are currently hospitalized for a minimum of 15 days after treatment. Clients can choose a short stay option where cats are discharged after 15 days provided certain conditions regarding radiation safety are met, including confining the cat to the house for an additional 8 days after discharge. Since most cats in the United Kingdom are indoor‐outdoor many owners are unwilling to do this and elect to leave their cats in the hospital until 23 days after RAI treatment (long stay) instead. Before 2012 the stringent radiation regulations that were in place meant that all cats had to remain in the hospital for 23 days after RAI treatment. Serum TT4 concentration was measured by an enzyme immunoassay (Immulite 1000 chemiluminescent TT4 assay) validated for use in cats. Grade of elevation of TT4 concentration at discharge was classified as mild (40‐100 nmol/L), moderate (100‐150 nmol/L), or severe (>150 nmol/L). Cats were coded as subsequent treatment success or failure. Treatment failure was defined as repeated documentation of TT4 concentration ≥40 nmol/L during follow‐up or additional treatment for hyperthyroidism, whether that was with repeated RAI treatment, surgery or reinstitution of medical therapy. Subsequent treatment success was defined as TT4 concentration <40 nmol/L measured at subsequent follow‐up without further medical, surgical, or RAI treatment of hyperthyroidism. For the purposes of this study, a TT4 concentration of less than 40 nmol/L was considered a treatment success, this included both euthyroid and hypothyroid cats.

The dose of ^131^I administered to each hyperthyroid cat was selected by the clinician in charge of the case (an ACVIM or ECVIM diplomate, or a resident working with them) based on a scoring system that included severity of clinical signs and TT4 concentration at the time of hospital admission (TT4 concentration before RAI treatment value), with medical or dietary treatment stopped 2 weeks before. Before September 2015 the doses administered were usually 111, 148, or 185 MBq (Table 1). After this date, an adjustment to the dosing schedule was made to include an additional lower dose of RAI (74 MBq) for cats with only mildly increased TT4 concentration and very mild or absent clinical signs (Table 2). A dose of 250 MBq was occasionally used, at the clinician's discretion, if the TT4 concentration before RAI treatment was exceptionally high, if thyroid cysts were suspected or if there was a very large palpable goiter. Scintigraphy was not routinely performed. Weight normalized RAI doses were calculated for each cat by dividing the dose (MBq) by bodyweight (kg).

**TABLE 1:** A scoring system was used to calculate the dose of radioactive iodine administered to each cat

Scoring system before 2015		
Score	Clinical signs	Serum total thyroxine concentration
1	Mild	<125 nmol/L
2	Moderate	125‐250 nmol/L
3	Severe	>250 nmol/L
**Total score**		**Dosage**
<3		111 MBq
4		148 MBq
5‐6		185 MBq

*Note*: A score was assigned to both TT4 concentration at the time of hospital admission and the clinical signs. These were then added together and on the basis of that figure the dose was given.

**TABLE 2:** The scoring system used to calculate the dose of radioactive iodine administered to each cat was modified in 2015

Scoring system after 2015		
Score	Clinical signs	Serum total thyroxine concentration
1	Very mild/none	<90 nmol/L
2	Mild	90‐125 nmol/L
3	Moderate	125‐250 nmol/L
4	Severe	>250 nmol/L
**Total score**		**Dosage**
2		74 MBq
3‐4		111 MBq
5‐6		148 MBq
7‐8		185 MBq

### 2.2 Statistical analysis

Statistical analysis was performed using a commercially available statistical software package (SPSS, Statistical Package for the Social Science, Software Packets for MacOS, Version 25; GraphPad Prism8, GraphPad Software for MacOS, San Diego, California). Numerical variables were assessed for normality by visual inspection of histograms and Shapiro‐Wilk test. Categorical data were compared using Pearson Chi‐squared test. Parametric Student's *t* test was used to compare normally distributed continuous data between 2 groups. Mann‐Whitney *U* test was used to compare non‐normally distributed continuous data. Factors that were individually associated with treatment outcome at *P* < .1 were then entered into binary logistic regression to build a multivariable model of treatment failure. The association between the TT4 concentration at discharge and failure of treatment was tested using receiver operating characteristic (ROC) curve analysis. The area under the receiver operating characteristic curve (AUROC) was calculated to evaluate the diagnostic performance of the TT4 concentration at discharge to predict failure of treatment. Youden's index was used to determine an ideal diagnostic cut‐off with adjustment based on expected clinical utility. Time to development of euthyroidism was depicted graphically using Kaplan‐Meier curves, with documentation of TT4 concentration <40 nmol/L defined as an event and cats censored if they died without documented euthyroidism, had repeat treatment with RAI, had thyroidectomy performed, or restarted medical management. If a cat was still alive but lost to follow‐up, it was censored on the date that TT4 concentration was last measured. Normally distributed data are reported as mean ± SD (range) and non‐normally distributed data are reported as median [25th, 75th percentile]. Statistical significance was defined as *P* < .05.

## 3 RESULTS

**FIGURE 1:**
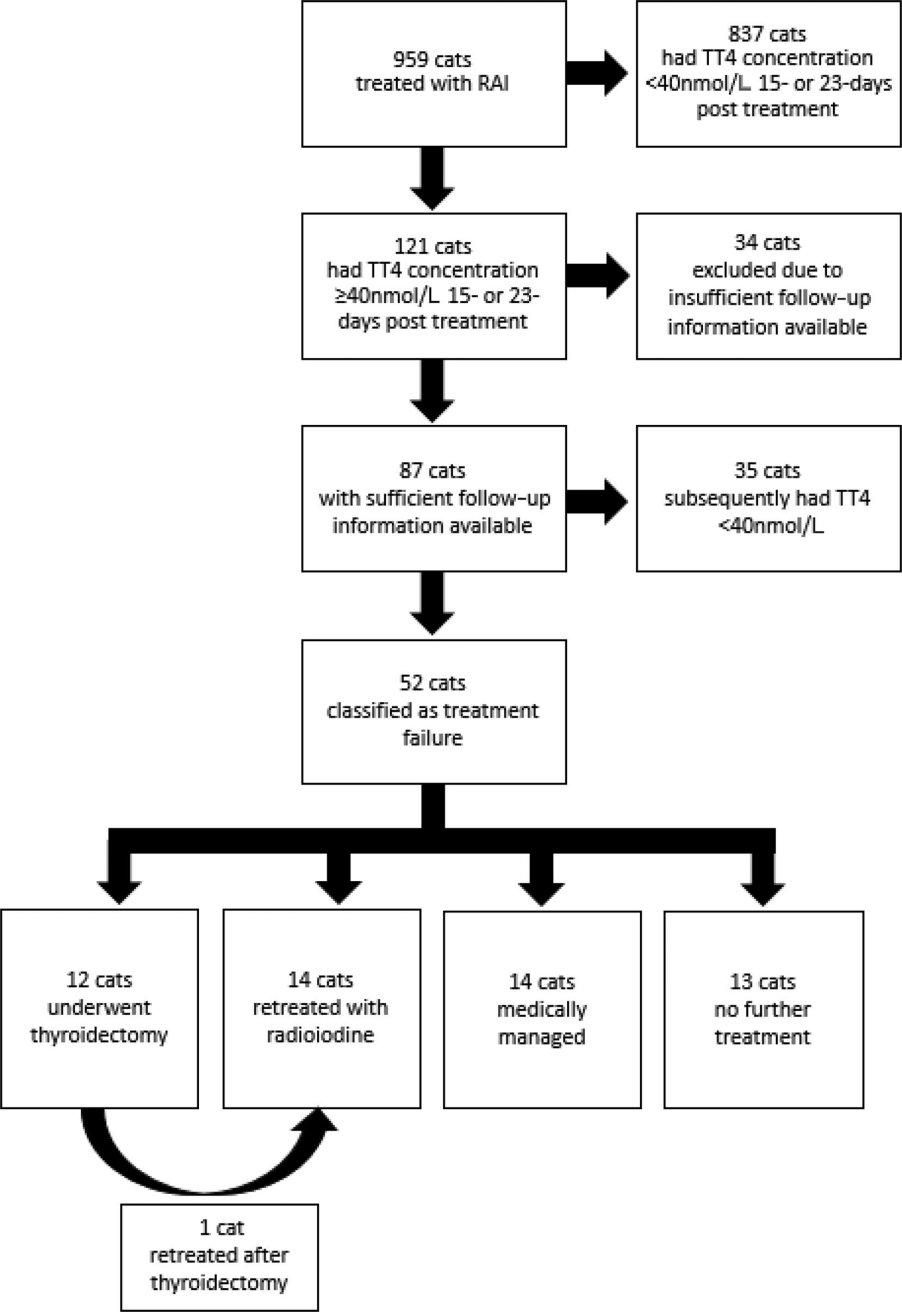
Details of 959 hyperthyroid cats treated with radioiodine including clinical outcomes for cats in which total thyroxine concentration remained elevated at discharge

Medical records of 959 cats that were treated with RAI from January 2007 to October 2018 were reviewed (Figure 1). Data completeness for the variables to be assessed were as follows; breed 100%, age 100%, sex 100%, bodyweight 99.8%, RAI dose 99.5%, TT4 concentration before RAI treatment 99.9%, TT4 concentration at discharge 99.9%, percentage change in TT4 concentration 99.9%, stay duration 100%. Breed was recorded for each cat but because of the low number of purebred cats, breed was classified as purebred and nonpurebred. Sex and neuter status was recorded for each cat, but because of the small number of intact cats, sex was coded as male or female. Eight hundred thirty‐seven (87.4%) cats had TT4 concentration < 40 nmol/L at the time of discharge and were considered to have been successfully treated and were not studied further. One hundred twenty‐one cats had TT4 concentration at discharge ≥40 nmol/L (94.9 [59.3, 156.0] nmol/L). There was no recorded TT4 concentration at discharge for 1 cat which was excluded from the logistic regression analysis, although this cat was documented to be euthyroid 1 month later. Of these 121 cats, 2 cats did not receive full dose of RAI due to misinjection. These cats were retreated with RAI and also excluded from analysis. Fifteen of the cats with TT4 concentration at discharge ≥40 nmol/L (70.6 [48.8, 113.0] nmol/L) were described in follow‐up visits to the referring vet as having clinical improvement with no biochemical documentation of euthyroidism. These cats were alive more than 1 year after treatment, had at least 2 revisits to their primary care practitioner, with no further medication for hyperthyroidism dispensed within the follow‐up period, and were documented to have either a stable weight or weight gain and no reported clinical signs of hyperthyroidism. However, further TT4 concentration testing was not undertaken (in spite of our recommendation that this be performed) and these cats were excluded from statistical analysis. Seventeen cats were lost to follow‐up. In total, 34 cats were excluded leaving 87 cats for further analysis. Fifty‐two (60%) cats were classified as treatment failure and 35 (40%) were subsequently classified as treatment success. As a total of the study sample treated, with adequate follow‐up, 52/925 (5.6%) cats were classified as treatment failure.

There were no significant differences in age at the time of presentation, sex, breed, stay duration, or dose of RAI for the cats with TT4 concentration >40 nmol/L at discharge that were subsequently classified as success and those were classified as failure. Summary statistics are presented in Table 3. Weight before RAI treatment approached significance with those classified as subsequent failure (3.6 [3.0, 4.3] kg) being lighter than those classified as subsequent success (4.2 [3.2, 5.3] kg; *P* = .05). Cats that were classified as treatment failure received higher dose per kg of bodyweight (50.7 [38.5, 62.1] MBq/kg) than those classified as subsequent success (38.6 [32.9, 47.4] MBq/kg; *P* = .005).

**TABLE 3:** Predictors of treatment failure in 87 cats with TT4 ≥40 nmol/L after single injection of radioiodine for treatment of hyperthyroidism

Variable		Failure (n = 52)	Success (n = 3S)	*P* value
Age (years)		12.4 [10.4, 14.0]	12.4 [11.0, 14.0]	.75
Breed	Purebred	1	1	.65
	Nonpurebred	51	34	
Stay duration	Long stay	24	16	.57
	Short stay	28	19	
Dose (MBq)	74	1	1	.07
	111	2	4	
	148	10	13	
	185	35	17	
	250	4	0	
Weight (kg)		3.6 [3.0, 4.3]	4.2 [3.2, 5.3]	.05
Weight normalized dose (MBq/kg)		50.7 [38.5, 62.1]	33.6 [32.9, 47.4]	.005
TT4 pretreatment (nmol/L)		383.8 [251.8, 513.8]	270.0 [211.0, 372.0]	.003
TT4 at discharge (nmol/L)		162.5 [100.75, 258.25]	62.3 [48.0, 98.1]	<.001
% change in TT4		−56.1 [−69.3, −34.0]	−72.3 [−81.8, −61.6]	<.001

Cats that were classified as treatment failure had significantly higher TT4 concentration before RAI treatment (383.8 [251.8, 513.8] nmol/L) than cats that became euthyroid (270.0 [211.0, 372.0] nmol/L; *P =* .003), higher TT4 concentration at discharge (162.5 [100.8, 258.3] nmol/L) than cats that became euthyroid (62.3 [48.0, 98.1] nmol/L; *P* < .001) and a lower percentage change in TT4 concentration (−56.1% [−69.3%, −34.0%]) than cats that became euthyroid (median −72.8% [−81.8%, −61.6%]; *P* < .001).

**FIGURE 2:**
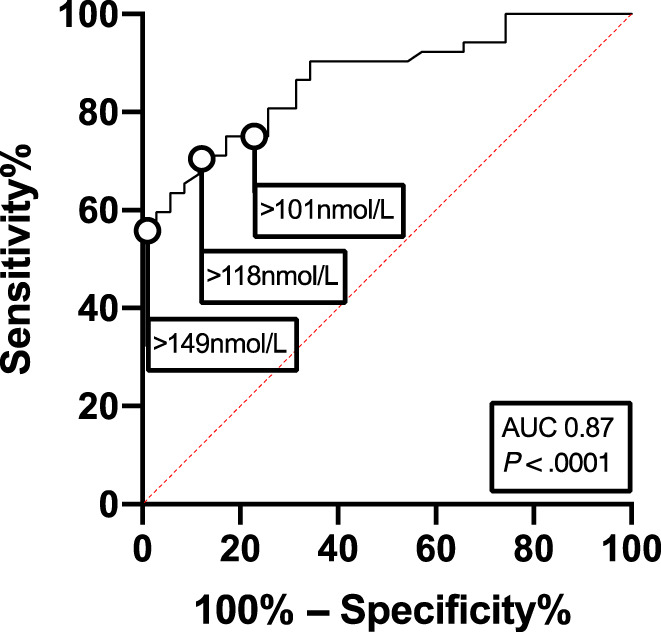
Receiver operator characteristic curve for use of serum total thyroxine concentration at discharge from hospital to predict treatment failure in 87 cats with TT4 ≥ 40 nmol/L after single injection of radioiodine. Area under the curve (AUC) is 0.87 [95% CI: 0.80‐0.94], *P* < .0001. An optimum cut‐off value, with the least amount of overlap between groups (optimal combination of sensitivity and specificity and highest Youden index) of 118 nmol/L predicted treatment failure with a sensitivity of 71.2% and a specificity of 88.6%. A clinically useful cut‐off of 101 nmol/L predicted treatment failure with a sensitivity of 75% and a specificity of 77%. The most specific cut off for predicting treatment failure was 149 nmol/L which predicted treatment failure with a sensitivity of 55.8% and a specificity of 100%. *P*, significance

In univariable logistic regression analysis, higher TT4 concentration before RAI treatment, higher TT4 concentration at discharge, and higher weight normalized RAI dose were positively associated with eventual treatment failure. Lower percentage change in TT4 concentration was negatively associated with eventual treatment failure. Further analysis of the data by multiple logistic regression with backward selection was then carried out. The variables included in the initial multivariable model to predict treatment failure in cats with elevated TT4 concentration after RAI treatment were TT4 concentration before RAI treatment, TT4 concentration at discharge, percentage change in TT4 concentration, weight, weight normalized RAI dose, and dose of RAI based on 3 categories (<120 MBq, 120‐175 MBq, >175 MBq). Total thyroxine concentration at discharge (*P* < .001) and weight normalized RAI dose (*P =* .04) were independently predictive of treatment failure. Receiver operating characteristic analysis of the multivariable model for treatment failure found an area under the curve (AUC) 0.9 (95% CI 0.83‐0.96; *P* < .001). Receiver operating characteristic analysis of TT4 concentration at discharge alone had an AUC 0.87 (95% CI 0.79‐0.94; *P* < .001; Figure 2). This revealed that TT4 concentration at discharge of >118 nmol/L maximized the sensitivity (71.2%) and specificity (88.6%) of predicting treatment failure. A cut‐off of 149 nmol/L had 100% specificity for predicting treatment failure. Of cats with TT4 concentration at discharge of 40‐100, 100‐150 and ≥150 nmol/L, treatment ultimately failed in 13/40 (32.5%), 11/19 (57.9%), and 28/28 (100%) respectively.

### 3.1 Follow‐up

**FIGURE 3:**
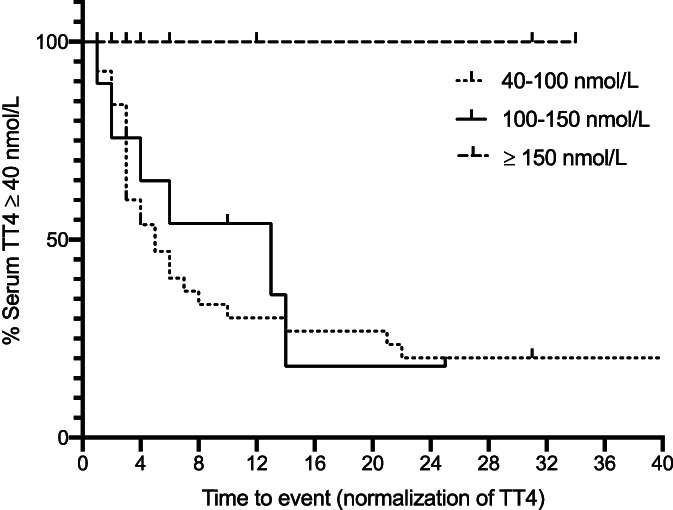
Kaplan‐Meier time‐to‐event curve for 87 cats with serum total thyroxine concentration (TT4) ≥40 nmol/L after single injection of radioiodine. Time to event is depicted with event referring to normalization of TT4 concentration (reduction to <40 nmol/L). Cats were allocated to 3 groups according to degree of elevation of TT4 at discharge from hospital after treatment with radioiodine (mild; TT4 40‐100 nmol/L, n = 40, % censored = 32.5%, moderate; TT4 100‐150 nmol/L, n = 19, % censored 58%, severe; TT4 ≥150 nmol/L, n = 28, % censored = 100%). Censored observations are indicated by a vertical line. Cats were censored if they died without documented euthyroidism, had repeat treatment with radioiodine, had thyroidectomy performed or restarted medical management. If a cat was still alive but lost to follow‐up without documentation of euthyroidism, it was censored on the date that TT4 was last measured. Median time to TT4 <40 nmol/L was 8 months [95% CI, 3‐13 months]

Follow‐up data for 52 cats classified as treatment failure is presented in Figure 1. Cats were censored for Kaplan‐Meier analysis at the time of first event. Seven of the cats had multiple treatments, for example, restarting medical management before retreatment with RAI and they were censored at the time of first treatment. Cats were censored either because they were euthanized for other reasons (n = 9), had thyroidectomy (n = 10), were retreated with RAI (n = 13), restarted medical management (n = 16), or were lost to follow‐up after multiple recordings of persistently elevated TT4 concentration (n = 4). In total, 35/87 cats had TT4 concentration <40 nmol/L without further treatment during the follow‐up period and were classified as a subsequent success. In 26/35 cats this was recorded within 6 months of discharge from hospital. Results of Kaplan‐Meier time‐to‐event analysis yielded an estimated median time to TT4 concentration <40 nmol/L of 8 months [95% CI, 3‐13 months]. These results are presented in Figure 3.

### 3.2 Repeat treatment with RAI

A total of 14 cats were retreated with RAI including 1 cat that underwent thyroidectomy before repeat treatment. These cats had TT4 concentration of 220.0 [126.5, 298.0] nmol/L before the second RAI treatment, which occurred 14‐1652 days after the first. After the second treatment with RAI TT4 concentration was 14.25 [5.1, 53.1] nmol/L at discharge. One cat had a very delayed repeat treatment (1652 days), this cat was clinically well immediately after treatment with a TT4 concentration of 51.7 nmol/L at discharge, and considered to be a treatment success at the time. However, clinical signs of hyperthyroidism recurred 4 years later, and this cat was retreated resulting in a TT4 concentration at discharge of 15 nmol/L. This cat remained well with normal TT4 concentration at the time of follow‐up. Second treatment dose ranged from 111 to 185 MBq in 12 cats treated for benign disease and 1000‐1100 MBq in 2 cats treated for presumed thyroid carcinoma based on thyroid scintigraphy or confirmed on histopathology. Ten of 14 cats had TT4 concentration <40 nmol/L after the second RAI dose, including both cats treated for presumed thyroid carcinoma. Four cats had elevated TT4 concentration at discharge from second treatment with RAI (TT4 concentration ≥40 nmol/L) although 2 of these cats later became euthyroid with no further treatment. In 1 cat that underwent thyroid scintigraphy before repeat RAI treatment, ectopic thyroid tissue suspicious for thyroid carcinoma was identified. Higher doses of RAI were not offered at our hospital at that time (2008) and the owner declined referral to another hospital for high dose RAI and the cat was treated at our hospital with 296 MBq RAI. Two months after repeat RAI treatment, this cat remained hyperthyroid (TT4 concentration 66.7 nmol) but clinical signs had markedly improved. The final cat remained hyperthyroid after second injection of RAI had thyroid scintigraphy performed before second RAI treatment which identified bilateral cervical thyroid uptake with no identified ectopic tissue. Retreatment with 185 MBq RAI was unsuccessful (TT4 concentration 124 nmol/L) and the owner opted to restart medical therapy. This cat was well controlled medically until the time of euthanasia 6 years later. In total, thyroid scintigraphy was performed on 4 cats before the second injection of ^131^I, including the 2 cats treated for presumed carcinoma and 2 of the cats described above that still had elevated TT4 concentration at the time of discharge after the repeated treatment.

### 3.3 Thyroidectomy

Twelve cats had thyroidectomy performed after treatment with RAI. Thyroid carcinoma was confirmed on histopathology in 5 of these cats and 5 cats had thyroid adenoma confirmed on histopathology. Results of histopathology for the remaining 2 cats (in which thyroidectomy was performed in the primary care practice) were not available for review. Four cats had TT4 concentration <40.0 nmol/L after thyroidectomy. Five cats were lost to follow‐up after thyroidectomy. Two cats restarted medical management for persistent hyperthyroidism after thyroidectomy including 1 of the cats that had been treated medically before surgery. The final cat was successfully treated with high dose RAI (1100 MBq) for treatment of thyroid carcinoma documented on histopathology after thyroidectomy, which was performed 2 months after initial unsuccessful RAI treatment (TT4 concentration before first RAI treatment 134.0 nmol/L, treated with 148 MBq RAI, TT4 concentration at discharge after first RAI treatment 120.0 nmol/L). Total thyroxine concentration at discharge after second RAI treatment was <5.1 nmol/L.

### 3.4 Restarting medical management

Fourteen cats were medically managed (methimazole, thiamazole, carbimazole) and were reported to be well controlled, however, 1 cat was euthanized 6 months after restarting medical management due to difficulties medicating.

### 3.5 Treatment failure with no further treatment

The remaining 13 cats that failed RAI treatment had no further treatment of hyperthyroidism. Four of these cats were still alive at the end of the study and reported to have adequately controlled clinical signs despite persistently elevated TT4 concentration and the owners declining to give any further treatment. During the follow‐up period for this group (3‐12 months) median lowest recorded TT4 concentration was 79.6 nmol/L [56.0, 93.0]. Nine of the cats that failed RAI treatment and had no further treatment of hyperthyroidism were euthanized for other reasons during the follow‐up period. Median time to euthanasia was 69 [21, 788] days. Before euthanasia, these cats also were reported to have adequately controlled clinical signs despite persistent hyperthyroidism.

## 4 DISCUSSION

Results of this study show that cats with elevated TT4 concentration at the time of hospital discharge after RAI treatment can subsequently become euthyroid. Cats with TT4 concentration at discharge <100 nmol/L might be good candidates for monitoring without additional treatment because based on the results of this study approximately two thirds will become euthyroid in time. However, this is unlikely to occur if TT4 concentration at discharge is greater than 150 nmol/L and early repeat treatment might be the preferred option. Repeat treatment was shown to have a good success rate in this study sample. Of the cats with TT4 concentration at discharge of 100‐150 nmol/L at discharge 8/19 (42.1%) subsequently became euthyroid and decision making in this category might depend on owner and clinician preference. Weight normalized RAI dose was found to be independently predictive of eventual treatment failure in this sample, with cats that failed treatment having higher weight normalized RAI doses. Area under the curve of ROC for multivariable model including TT4 concentration at discharge and weight normalized dose was marginally higher than AUC of ROC analysis of TT4 concentration at discharge alone. However, the increased complexity of combining 2 (in effect 3) different parameters could not be justified because this would be much harder to use in the clinic. In contrast to what was anticipated at the inception of this study, the timing of measurement of TT4 concentration after treatment (ie, 15 or 23 days after RAI treatment) did not alter the probability of the treatment eventually being classified as a success. It was thought that if the blood sample had been collected earlier, then the cat would be more likely to become euthyroid during follow‐up, but this was not the case.

Cats that remain hyperthyroid after treatment with RAI are a clinical challenge. Some owners might opt to restart medical management; however, 48.6% of owners struggle to administer tablets to their hyperthyroid cats,^12^, ^13^ and this proportion might be even greater in cats presented for RAI treatment. In this study, 1 cat that remained hyperthyroid after RAI and restarted medical management was euthanized 6 months later due to difficulties administering medication. In addition, some cats are referred for RAI treatment because of the development of serious side‐effects after treatment with thioureas (eg, facial excoriations, blood dyscrasias) and in these cats resuming medical treatment would be inappropriate.^14^

Twelve cats underwent thyroidectomy for management of persistent hyperthyroidism after RAI treatment, but this only resulted in euthyroidism in 4 of the cats. This treatment might have been chosen because it could be performed at lower cost than RAI therapy. However, recurrence of hyperthyroidism is common in cats having thyroidectomy performed in first‐opinion practice, even if cats are initially hypothyroid after the surgery.^15^ The presence of ectopic hyperfunctional thyroid tissue is reported in 3.9%‐23% of cats undergoing thyroid scintigraphy indicating that surgical management would not be curative in these cases.^16^, ^17^ One argument in favor of surgical treatment is to obtain tissue for histopathological examination where thyroid carcinoma is suspected. Thyroid carcinoma was confirmed in this study in 5/10 cats where histopathology was performed, presumably because of a selection bias towards surgery in cases where this diagnosis was suspected after the failure of initial RAI treatment. This was probably partly responsible for the low success rate of surgery in this study.

The success rate of repeat treatment with RAI after initial treatment failure has not been reported, although isolated case reports exist.^18^ Results of our study are supportive of repeat treatment with RAI because this was successful in 10/12 cats treated for benign disease with standard (<250 MBq) doses of RAI, as well as both cats receiving a high (>1000 MBq) dose for treatment of thyroid carcinoma. Although these results are encouraging this might reflect careful case selection of cats that were considered likely to respond to standard doses.

There are several limitations to this study. The retrospective nature of the study meant that complete follow‐up was not available for all cats. In cases defined as treatment success, it was presumed that TT4 concentration never rose above the reference range again, although this was not consistently proven in every case. In many cases the recommendations for follow‐up testing were not followed. In recent years we have included measurement of TT4 and thyroid stimulating hormone (TSH) 6 months after treatment as part of our RAI “package” in order to detect hypothyroidism and have followed up on several hundred cats in this manner. Anecdotally, we can report that none of the cats with TT4 <40 nmol/L at discharge have been hyperthyroid at 6 months. In some cases, the infrequency of repeat TT4 concentration measurement might have been because the cats were clinically well, either because the hyperthyroidism had resolved or because it was much less severe than before RAI treatment. This might have resulted in overestimation of the median time interval for normalization of TT4 concentration in the Kaplan‐Meir survival analysis, and the wide 95% confidence intervals. Furthermore, not all reassessments of TT4 concentration were performed in a reference laboratory or using the same methodology. Because of the large retrospective nature of this study, it was not possible to provide details of measurement methods in every case. It is recommended that the same assay technique and laboratory are used when monitoring TT4 concentration and a recent study has suggested significant differences between results using different methods, particularly at higher hormone concentrations.^19^ In addition, when TT4 concentration was reassessed, this was sometimes tested as part of investigations when the cat was presented for another illness, meaning that TT4 concentration might have been falsely low in some cases, due to nonthyroidal illness.

Thyroid carcinoma was definitively diagnosed in 5/959 (0.5%) cats in this study sample. The prevalence of thyroid carcinoma in hyperthyroid cats is 1%‐3%,^20^ but cats were only included in the present study if they were initially thought to have, and were being treated for, benign thyroid disease. For much of the period of the study cats known to have thyroid carcinoma had to be referred elsewhere for treatment because our license did not permit us to administer high doses (>300 MBq) of RAI. It is possible that more cats in this study sample had thyroid carcinoma which was not detected clinically but successfully treated with (conventional dose) RAI. Thyroid scintigraphy is not routinely performed before RAI treatment in our clinic although it is offered for cats that have failed initial RAI treatment, or in any cat where there is a suspicion of thyroid carcinoma based on physical examination (large, irregular, or fixed thyroid gland). There is no significant relationship between thyroid volume and persistent hyperthyroidism after treatment with RAI,^21^ however, cats with very large cystic goiters have a low success rate for RAI treatment.^22^

Although the upper limit of the reference range for TT4 concentration in our laboratory is 65 nmol, TT4 concentration at discharge of ≥40 nmol/L was considered inappropriately high in this study sample. The modal TT4 concentration measured 19 days after RAI treatment is reported as 10.0 nmol/L (IQR 10.0‐13.6 nmol/L).^18^ Furthermore, older cats with comorbidities are expected to have serum TT4 concentration lower than 40 nmol/L.^23^

In this study sample, 121/959 (12.5%) cats had elevated TT4 concentration at the time of discharge from the hospital. Adequate follow‐up was available for 87 of these cats and the overall, treatment failure rate of 5.6% (52/925) after a longer period of follow‐up was similar to that reported previously.^3^, ^8^, ^9^, ^24^

Nine cats had an increase in TT4 concentration at the time of discharge from hospital after RAI treatment. This might be due to the fact that although it is recommended to discontinue medical treatment of hyperthyroidism for 2 weeks before treatment with RAI, some cats might have continued medical management due to clinician or owner concerns. Alternatively, their TT4 concentration might have been suppressed initially due to transient nonthyroidal illness or laboratory errors could have occurred.

In conclusion, cats with elevated TT4 concentration at the time of discharge from hospital after RAI treatment can later become euthyroid and the degree of elevation of TT4 concentration at discharge is a predictor of ultimate treatment failure, however, this prediction cannot be improved by also considering other parameters such as how high the TT4 concentration was before RAI treatment, or how long after treatment the sample was collected. Cats with mild elevation of TT4 concentration (40‐100 nmol/L) might be good candidates for monitoring as they have a good chance of becoming euthyroid. In contrast, no cats with severe elevation of TT4 concentration (≥150 nmol/L) at the time of discharge from hospital after RAI treatment subsequently became euthyroid in this study and these cats are good candidates for retreatment with RAI.

## CONFLICT OF INTEREST DECLARATION

Authors declare no conflict of interest.

## OFF‐LABEL ANTIMICROBIAL DECLARATION

Authors declare no off‐label use of antimicrobials.

## INSTITUTIONAL ANIMAL CARE AND USE COMMITTEE (IACUC) OR OTHER APPROVAL DECLARATION

Approved by the Royal Veterinary College clinical research ethical review board (URN: SR2019‐0210).

## HUMAN ETHICS APPROVAL DECLARATION

Authors declare human ethics approval was not needed for this study.
